# SLF2 Interacts with the SMC5/6 Complex to Direct Hepatitis B Virus Episomal DNA to Promyelocytic Leukemia Bodies for Transcriptional Repression

**DOI:** 10.1128/jvi.00328-23

**Published:** 2023-06-20

**Authors:** Qiyan Yao, Bo Peng, Cong Li, Xuelei Li, Mingyi Chen, Zhongmin Zhou, Dingbin Tang, Jiabei He, Yumeng Wu, Yinyan Sun, Wenhui Li

**Affiliations:** a Graduate School of Peking Union Medical College, Chinese Academy of Medical Sciences, Beijing, China; b National Institute of Biological Sciences, Beijing, China; c School of Life Sciences, Tsinghua University, Beijing, China; d Tsinghua Institute of Multidisciplinary Biomedical Research, Tsinghua University, Beijing, China; University of Southern California

**Keywords:** hepatitis B virus, cccDNA, SLF2, PML bodies, SMC5/6 complex

## Abstract

Hepatitis B virus (HBV) chronically infects approximately 300 million people worldwide, and permanently repressing transcription of covalently closed circular DNA (cccDNA), the episomal viral DNA reservoir, is an attractive approach toward curing HBV. However, the mechanism underlying cccDNA transcription is only partially understood. In this study, by illuminating cccDNA of wild-type HBV (HBV-WT) and transcriptionally inactive HBV that bears a deficient HBV X gene (HBV-ΔX), we found that the HBV-ΔX cccDNA more frequently colocalizes with promyelocytic leukemia (PML) bodies than that of HBV-WT cccDNA. A small interfering RNA (siRNA) screen targeting 91 PML body-related proteins identified SMC5-SMC6 localization factor 2 (SLF2) as a host restriction factor of cccDNA transcription, and subsequent studies showed that SLF2 mediates HBV cccDNA entrapment in PML bodies by interacting with the SMC5/6 complex. We further showed that the region of SLF2 comprising residues 590 to 710 interacts with and recruits the SMC5/6 complex to PML bodies, and the C-terminal domain of SLF2 containing this region is necessary for repression of cccDNA transcription. Our findings shed new light on cellular mechanisms that inhibit HBV infection and lend further support for targeting the HBx pathway to repress HBV activity.

**IMPORTANCE** Chronic HBV infection remains a major public health problem worldwide. Current antiviral treatments rarely cure the infection, as they cannot clear the viral reservoir, cccDNA, in the nucleus. Therefore, permanently silencing HBV cccDNA transcription represents a promising approach for a cure of HBV infection. Our study provides new insights into the cellular mechanisms that restrict HBV infection, revealing the role of SLF2 in directing HBV cccDNA to PML bodies for transcriptional repression. These findings have important implications for the development of antiviral therapies against HBV.

## INTRODUCTION

Hepatitis B virus (HBV) is a DNA virus that infects approximately 300 million people worldwide (1, 2). While antivirals such as nucleos(t)ide analogs can reduce viral load by inhibiting HBV reverse transcription, they fail to cure chronic HBV infection due to the persistence of the viral reservoir, the covalently closed circular DNA (cccDNA), in hepatocytes (3–5). Upon entry into the host cell via cellular receptor sodium taurocholate cotransporting polypeptide (NTCP) (6), HBV genome (relaxed circular DNA [rcDNA]) is delivered to the nucleus and repaired into the cccDNA, which is packaged as a minichromosome and serves as the template for all subsequent viral mRNA transcription (7, 8). Developing therapeutics to permanently silence cccDNA transcription has been considered an attractive approach for HBV treatment (5, 9, 10).

HBV cccDNA transcription is regulated by host factors and the viral X protein (HBx) (11–13). HBx is essential for sustaining HBV cccDNA transcription activity (14, 15). The interactions of HBx with host proteins have been widely investigated in studies seeking to determine their various roles in HBV infection. A well-defined function of HBx is that it hijacks the damage-specific DNA binding protein 1 (DDB1) to degrade the structural maintenance of chromosome 5/6 (SMC5/6) complex (16, 17), which comprises SMC5, SMC6, and NSE1 to 4 (18). Transcription of the X-minus HBV (HBV-ΔX) variant is repressed in virus-infected hepatocytes (16, 17, 19). The SMC5/6 complex thus functions as a host restriction factor that is antagonized by HBx.

Previous studies reported that the SMC5/6 complex colocalizes with scaffold proteins of promyelocytic leukemia (PML) bodies (PML and SP100) (20). PML bodies are membraneless nuclear bodies in the nucleus, and more than 80 proteins have been reported to constitutively reside in—or be transiently recruited to—PML bodies (21). PML bodies function in multiple biological processes, such as DNA damage response, transcriptional regulation, and apoptosis (22, 23). Multiple studies have shown the antiviral activity of PML bodies against various viruses, and consequently, viruses evolved countermeasures to antagonize host restriction by degrading proteins within PML bodies to promote their transcription activity and propagation (24–29). In wild-type HBV infection, PML knockout increased viral RNA levels (30); depletion of PML and SP100 was also shown to alter the distribution of SMC6 in the nucleus and to stimulate the transcription of HBV-ΔX, suggesting that PML bodies are involved in host restriction of HBV transcription by the SMC5/6 complex (20, 31). However, the molecular mechanism underlying SMC5/6-suppressing machinery assembly for HBV cccDNA silencing is unclear.

In this study, we show that transcriptionally silenced HBV-ΔX cccDNA colocalizes with PML bodies using HBV DNA fluorescent *in situ* hybridization (FISH). Using a targeted small interfering RNA (siRNA) knockdown screen focusing on proteins associated with PML bodies, we identified SMC5-SMC6 localization factor 2 (SLF2) as a host restriction factor for HBV transcription. SLF2 interacts with SMC5/6 complex through residues 590 to 710 and recruits SMC5/6 complex to PML bodies through the disordered region at its N terminus. In the presence of the SMC5/6 complex, SLF2 directs cccDNA to PML bodies. The SMC5/6-binding region of SLF2 is necessary for HBV transcription repression and the colocalization of transcriptionally silenced cccDNA with PML bodies. Our results show that SLF2 interacts with the SMC5/6 complex to direct HBV cccDNA to PML bodies for transcriptional repression.

## RESULTS

### Transcriptionally silenced HBV cccDNA colocalizes with PML bodies.

**FIG 1:**
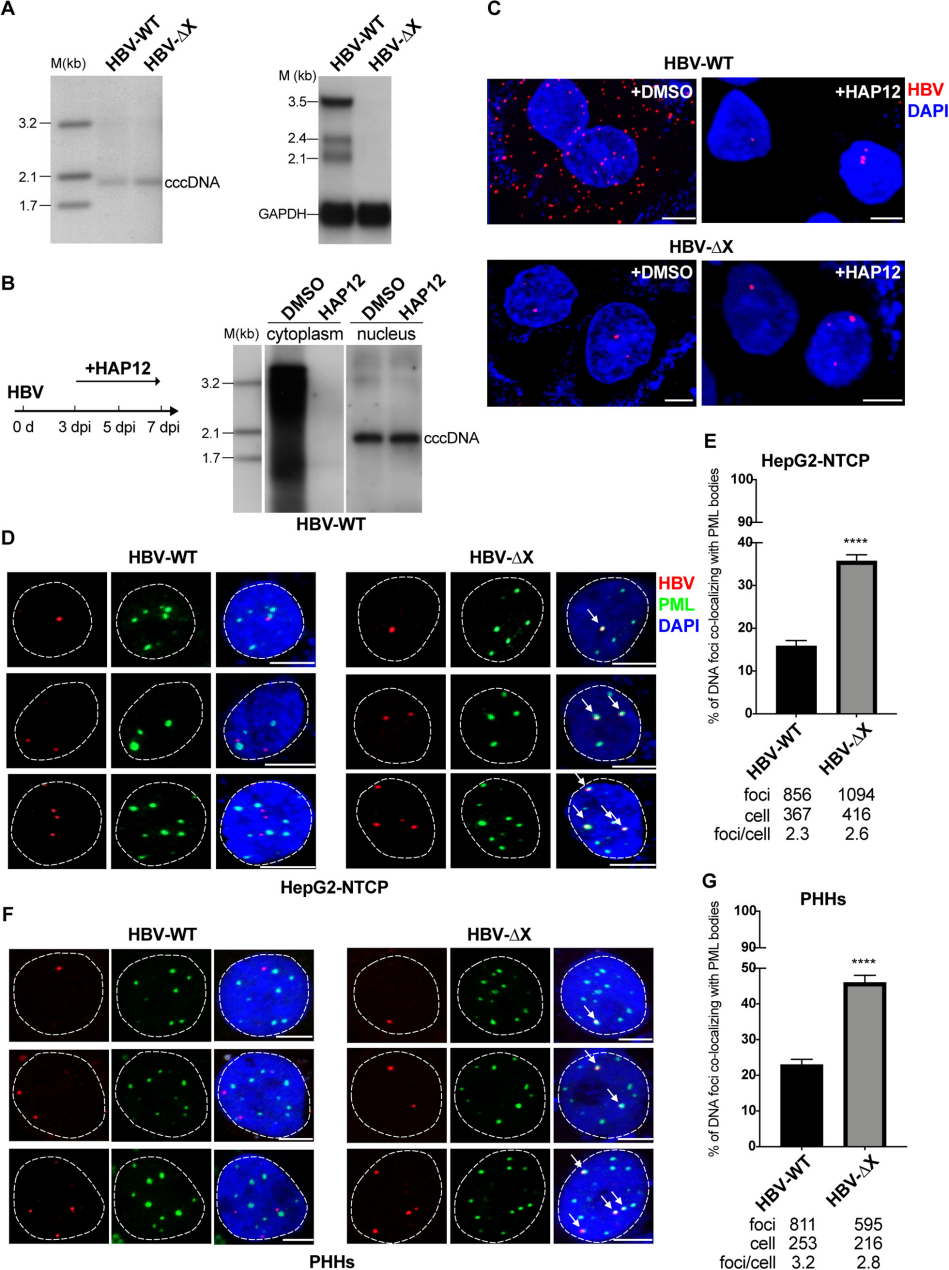
Colocalization of transcriptionally silenced HBV cccDNA with PML bodies. (A) Transcription of HBV is silenced in the absence of HBx. HepG2-NTCP cells were inoculated with wild-type HBV (HBV-WT) or X-minus HBV (HBV-ΔX). HBV cccDNA was extracted using the Hirt method at 7 dpi and analyzed by Southern blotting. The HBV RNA was analyzed by Northern blotting. (B and C) DNA FISH foci in the nucleus are mainly HBV cccDNA with HAP12 treatment. HepG2-NTCP cells were inoculated with HBV-WT or HBV-ΔX and treated with 5 μM HAP12 or dimethyl sulfoxide (DMSO) from 3 dpi to 7 dpi. The cytoplasm and nucleus were separated and subjected to DNA extraction. HBV-WT DNA was analyzed by Southern blotting (B). Cells were fixed and subjected to HBV DNA FISH (C). (D and E) Colocalization analysis of HBV-WT DNA and HBV-ΔX DNA with PML bodies in HepG2-NTCP cells. HepG2-NTCP cells were inoculated with HBV-WT or HBV-ΔX. Cells were treated with 5 μM HAP12 from 3 dpi to 7 dpi and fixed at 7 dpi. HBV DNA was stained using probes targeting full-length HBV genome, and PML bodies were stained using PML antibody. Representative confocal images show the HBV DNA (red) and the PML bodies (green) (D). The white arrows indicate the HBV DNA colocalizing with PML bodies. The distance (*d*) of DNA foci to PML bodies was measured using Imaris software. The colocalization ratio was calculated using DNA foci colocalizing with PML bodies (*d* ≤ 0 μm) divided by total DNA foci. Total focus numbers, total cell numbers, and foci per cell analyzed were summarized from three independent experiments. (E) Statistical analysis of the colocalization ratio of HBV DNA with PML bodies in HBV-WT- and HBV-ΔX-infected cells. The results are the summaries of three independent experiments. (F and G) Colocalization analysis of HBV-WT DNA and HBV-ΔX DNA with PML bodies in PHHs. PHHs were inoculated with HBV-WT or HBV-ΔX. Cells were treated with 5 μM HAP12 from 3 dpi to 7 dpi and fixed at 7 dpi. Representative confocal images show the HBV DNA (red) and the PML bodies (green) (F). Statistical analysis of the colocalization ratio of HBV DNA with PML bodies in HBV-WT- and HBV-ΔX-infected PHHs (G). The results are the summaries of two independent experiments. The *P* value was calculated using Fisher’s exact test. Error bar shows the standard error of the mean (SEM) calculated according to Bernoulli distributions. The scale bar represents 5 μm. ****, *P* < 0.0001.

Previous studies have reported that the HBx protein degrades the host restriction factor SMC5/6 complex to activate HBV transcription (16). To study the regulation of HBV cccDNA transcription in the presence or absence of HBx, we utilized recombinant wild-type HBV (HBV-WT) and an X gene-deficient HBV variant (HBV-ΔX), which was generated by introducing a premature termination codon to the X gene (19). The HBV-ΔX established a similar cccDNA level in host cells to that of HBV-WT but could only generate X-related transcripts (32). As expected, viral transcription was significantly repressed in the cells infected with HBV-ΔX (Fig. 1A).

HBV cccDNA was proposed to colocalize with PML bodies through its interaction with the SMC5/6 complex (20, 31). However, experimental data was only limited to the colocalization of PML bodies with the SMC5/6 complex. To directly investigate and quantify cccDNA localized in PML bodies, we used a previously reported HBV DNA FISH approach to detect cccDNA in HepG2-NTCP cells infected by HBV-WT (19). To minimize the signal of HBV DNA replicative intermediates (mainly the relaxed circular DNA), cells were treated with a capsid assembly inhibitor (HAP12) that can interrupt the accumulation of HBV rcDNA (33). HAP12 treatment had no impact on the cccDNA content but did result in drastic reductions of the levels of HBV replicative intermediates in the cytoplasm and nucleus as measured by Southern blotting and HBV DNA FISH (Fig. 1B and C). We stained PML bodies and HBV cccDNA, and found that approximately 36% of HBV DNA foci in the nucleus colocalized with PML bodies in the HBV-ΔX-infected cells in comparison to that of 16% in the HBV-WT-infected cells (Fig. 1D and E). A similar pattern (46% for HBV-ΔX versus 23% for HBV-WT) was observed in primary human hepatocytes (PHHs), arguing against cell line-specific processes for the colocalization of cccDNA with PML bodies (Fig. 1F and G). Together, these results show a functionally relevant colocalization of transcriptionally silenced cccDNA with PML bodies.

### An siRNA knockdown screen identifies SMC5-SMC6 localization factor 2 as a host restriction factor for HBV transcription.

**FIG 2:**
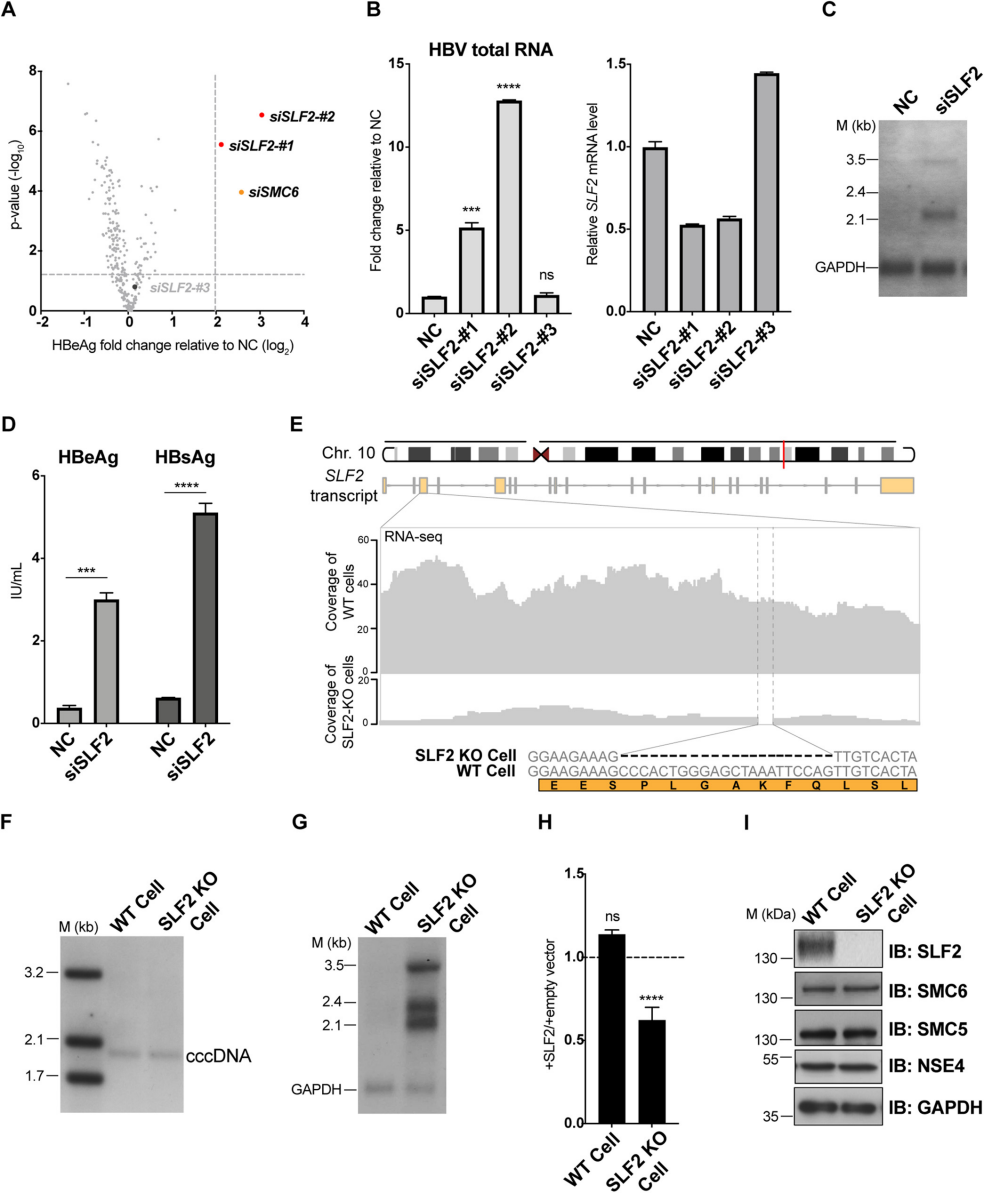
A knockdown screen identifies SMC5-SMC6 localization factor 2 as a cellular factor repressing HBV transcription. (A) Targeted siRNA screen for host factors repressing HBV infection. HepG2-NTCP cells were inoculated with HBV-ΔX. Cells were reseeded to 96-well plates at 3 dpi, and siRNAs targeting PML body-related proteins were transfected into infected cells. The HBeAg level in the supernatant was measured at 8 dpi. siRNA targeting SMC6 was used as the positive control. The horizontal dashed line represents a *P* value of 0.05; the vertical dashed line represents a fold change of 4. The volcano plot shows the HBeAg value fold change over NC and *P* value (*n* = 4). (B) Validation of putative host restriction factors. Cells were treated in the same manner as in panel A, and HBV total RNA was analyzed by qPCR (left panel). siRNA knockdown efficiency was analyzed, and GAPDH was taken as the reference gene (right panel). (C and D) Knockdown of SLF2 rescued viral transcription of HBV-ΔX in PHHs. PHHs were transfected with siRNA at 6 h after seeding. Cells were then inoculated with HBV-ΔX at 16 h after siRNA transfection and harvested at 11 dpi. HBV RNA was analyzed by Northern blotting (C), and HBeAg and HBsAg were measured (D). (E) Validation of SLF2 knockout single clonal cells. The red line indicates the gene locus of *SLF2* on chromosome 10. SLF2 knockout single clonal cells were identified by T-vector Sanger sequencing. The 22-bp nucleotide deletion form of the knockout clonal cells used in this paper is displayed. RNA-seq analysis of WT versus SLF2 knockout HepG2-NTCP cells shows the coverage of the third exon of *SLF2* transcripts in wild-type (HepG2-NTCP-Cas9, WT) (up) and SLF2 knockout (KO) HepG2-NTCP cells (down). The gap highlighted by the dashed line indicates the deleted sequence in SLF2 knockout HepG2-NTCP cells. (F) Analysis of the cccDNA level in WT or SLF2 KO HepG2-NTCP cells. The indicated cells were inoculated with HBV-ΔX. HBV cccDNA was extracted using the Hirt method at 7 dpi and analyzed by Southern blotting. (G) HBV-ΔX was actively transcribed in SLF2 KO HepG2-NTCP cells. WT or SLF2 KO HepG2-NTCP cells were inoculated with HBV-ΔX. HBV transcripts were detected by Northern blotting at 7 dpi. (H) Expression of SLF2 resulted in repression of HBV transcription in SLF2 knockout HepG2-NTCP cells. WT or SLF2 KO HepG2-NTCP cells were inoculated with HBV-ΔX, and the cells were transduced with lentivirus-expressing SLF2 or empty vector at 3 dpi. HBeAg in supernatant was measured at 10 dpi and is shown as the group complemented with SLF2/group complemented with empty vector. (I) Protein levels of SMC5, SMC6, and NSE4 in WT or SLF2 KO HepG2-NTCP cells. WT or SLF2 KO HepG2-NTCP cells were inoculated with HBV-ΔX. Cells were lysed at 8 dpi. Protein levels were analyzed by Western blotting. The *P* value was calculated using one-way analysis of variance (ANOVA) (B and D) and two-way ANOVA (H). The error bar shows the standard deviation (SD). ns, not significant; NC, negative control; ***, *P* < 0.001; ****, *P* < 0.0001.

PML bodies have been suggested to contain many proteins, including the SMC5/6 complex, and to act as reservoirs for these proteins (23). To identify host factors involved in recruiting HBV cccDNA to PML bodies, we utilized an siRNA library containing 273 siRNAs targeting 91 proteins that have been associated with PML bodies in previous studies (21, 34) (Table 1). We transfected HepG2-NTCP cells infected with HBV-ΔX with individual siRNAs from the library and measured hepatitis B e-antigen (HBeAg) levels in the supernatant at 8 days postinfection. Transfection with siRNAs targeting SMC5-SMC6 complex localization factor 2 (SLF2) resulted in significant activation of HBV-ΔX transcription (Fig. 2A and B), suggesting that SLF2 plays a role in the transcriptional repression of HBV. We therefore focused on SLF2 to investigate its potential role in HBV repression.

**TABLE 1:** siRNA sequence targeting PML body-related proteins

Gene symbol	siRNA no. 1	siRNA no. 2	siRNA no. 3
RUNX1	GCUGAGCUGAGAAAUGCUAtt	GGCAGAAACUAGAUGAUCAtt	AGAACCAGGUUGCAAGAUUtt
ATR	UUGUAGAAAUGGAUACUGAtt	GAGCCGAUUUUUAAGUCAAtt	GAUGAGUAUGCAAAAUUUAtt
ATRX	CAAUGGUAUUGCUACAUUUtt	AGAUAAAAGUUGACAGUGAtt	CAGCGUAUUUGAGAAUUUAtt
BACH2	GAUAUUCUCUGUGACGUGAtt	CCUUUUCCUGUAGAUCAAAtt	CAAGAAUGUCUAUAAUGCAtt
BLM	CCCACUACUUUGCAAGUAAtt	GGAUGUUCUUAGCACAUCAtt	GAUAUCUUCCAAAACGAAAtt
ARID3A	AGAUCAACGGCAUCAUGUAtt	GGACACCUGUGAACCGCAUtt	GCAUCAGCAUGUCGGUGGAtt
CREBBP	GAAUCUUUCCCAUAUCGAAtt	CAGCUAUCAGAAUAGGUAUtt	GGAUAUUGCUGUGGACGCAtt
CHFR	GAAUUGUAGUGGAUGAAAAtt	GCAGUGUCCUGAGUACAGAtt	GGAAUAAAAUCACUCAAGAtt
CHEK2	GAUCAGUCAGUUUAUCCUAtt	GGCACGUUUUACGACAGAAtt	GCACUGUCACUAAGCAGAAtt
CCNT1	CGACCCAGACAAUAGACUAtt	CCUUGUUUCUAGCAGCUAAtt	GCCCAUAGAGGGUUCAGAAtt
DAXX	GCUCUAUGUCUACAUCAAUtt	CAUCGUUACUGUCAGAAGAtt	CUACAGAUCUCCAAUGAAAtt
DHX9	GAGUGUAACAUCGUAGUAAtt	CCCUGUCACUUGUCAGACAtt	GGUUGAAGCUUACUCCGGAtt
EIF3E	CAAACAAGGAUGUUCGAAAtt	GGAAUUAUUACAAGGUAAAtt	GGAAGACCUUACACGGUUAtt
EIF4E	CAAAGAUAGUGAUUGGUUAtt	GCUAAUUACAUUGAACAAAtt	GGUAUACAAGGAAAGGUUAtt
ELF4	GCGGAAGUCUUACUCAAUAtt	CCAUCUAUCUGUGGGAGUUtt	GCGAUAUCCUGGAUGAGAAtt
NBN	GCAAGGUCUUAGACCUAUUtt	GAUAAUUCUAAGUAUGGUAtt	GGAAGACUCACUAUGGUCAtt
FOXL2	CGAAGUUCCCGUUCUACGAtt	GCGAAGACAUGUUCGAGAAtt	ACCAGUACAUCAUCGCGAAtt
USP7	GGGCGAGCAUUUUCGAGAAtt	GGAAUGACAUGUACGAUGAtt	GCAGUUGGUGGAGCGAUUAtt
TP53	GUAAUCUACUGGGACGGAAtt	GAAAUUUGCGUGUGGAGUAtt	GGUGAACCUUAGUACCUAAtt
TP73	GCAAUAAUCUCUCGCAGUAtt	CCACCAUCCUGUACAACUUtt	ACCACUUUGAGGUCACUUUtt
PSME3	CAAUCUUAAACAUCCAUGAtt	CCAGGAUAGAAGAUGGAAAtt	CCAGAUUUCUAGAUAUUAUtt
PAWR	GAUAUAACAGGGAUGCAAAtt	AGAUAUUCUCGAACAGAUAtt	GAAUGAAGCUGUAAACUUAtt
PIAS4	AGGUGGAGUUGAUCCGGAAtt	GGAGUAAGAGUGGACUGAAtt	CGGAAAGUACUUAAACGGAtt
PML	GAUGCAGCUGUAUCCAAGAtt	GGAGCAGGAUAGUGCCUUUtt	GGCAGAUUGUGGAUGCGCAtt
PRMT1	GCAACUCCAUGUUUCAUAAtt	CGAGGACCGGCAGUACAAAtt	GCCUGCAAGUGAAGCGGAAtt
PTMA	UGACCAUGUUCAUUAUAAUtt	GGUUUGUAUGAGAUGGUUAtt	GUAGUUGGUUUGUAUGAGAtt
PTEN	GCAUACGAUUUUAAGCGGAtt	CACCGCAUAUUAAAACGUAtt	GGUUUUCGAGUCCUAAUUAtt
RAD50	GGUAGACUGUCAUCGUGAAtt	GGAAUAGACUUAGAUCGAAtt	GCAGUUGUCCAGUUACGAAtt
RBCK1	AGAUCGUGGUACAGAAGAAtt	AGUGCGCCCUGAUAUGACAtt	GCCUUCAGCUGUCAGCUUUtt
RECQL4	GGCUCAACAUGAAGCAGAAtt	CCUCGAUUCCAUUAUCAUUtt	CCCAAUACAGCUUACCGUAtt
TRIM27	GCUGAACUCUUGAGCCUAAtt	CAAAAAUGUCUAUUCUUGAtt	GCAGCUGUAUCACUCCUUAtt
RPAIN	GAAGCUUCGUGCCUGUUUAtt	ACAGCUUUCUAGUUCAAGAtt	AGUGGAGAAUUGUCCAGAAtt
RNF4	GUCUCAUCGUUUCCACAGAtt	CCUCACUUGUGAAUCUUUAtt	GUAUAUGUGACUACCCAUAtt
RPA1	CAAGCACUAUCAUUGCGAAtt	GCAUGAUCCUGUCAGUAAAtt	GGUGUAUUAUUUCUCGAAAtt
RPA2	GGAGAGCACCUAUCAGCAAtt	GCAGAAUAACUGGAUCUAAtt	GCACCUUCUCAAGCCGAAAtt
SAP25	GCAGUUCUCAGGGUGCUGAtt	AAUAAAACAGUGUUGGUUUtt	GUAUGAAGCCAAAGCAGGAtt
SATB1	GGCUCGUAUCAACACCUAUtt	CGAAUAUACCAGGACGAAAtt	GCAUUGCUGUCUCUAGGUUtt
SENP1	GCAUUUCGCCUGACCAUUAtt	CACAGUGUAUAUUCCCUAUtt	GAGGCGACAUGUUAGUACAtt
SENP2	GCCUAUUCAUCGGAAGGUAtt	GAAAGAGAGAAGUACCGAAtt	GAAUAAGUGACUAUCCAAAtt
SETDB1	GGACAAUGCAGGAGAUAGAtt	CAACCAGACAUAUAGAUCAtt	GCCGUGACUUCAUAGAGGAtt
SIRT1	GUAAGACCAGUAGCACUAAtt	GGCUUGAUGGUAAUCAGUAtt	GGGUCUUCCCUCAAAGUAAtt
ZCCHC12	CCCUUUAUGUGAUCCGUUUtt	GGAUUUUCUCAGGAUGUAUtt	GCGACCAACCCUAACCUAAtt
SP100	GCAUCUGACAUAAUAGUCAtt	GAGAAGGCAUAGAUCUAAAtt	GACUGAAAGUAGUCAAGCAtt
SP110	CCAAUCUGGUGACGAUUUAtt	GGAUGGAACUUGGUUAACAtt	GCCUCAUCUAGACACGGAAtt
SP140	GAUUUAAACGAGAUUUACAtt	GAGCAGAUGUAUGAACAUUtt	ACUUCAGGAUGGUCGCAGAtt
SPTBN4	AGCAGCACAUCAAAAAGAAtt	CCACAAGCUGCAUAAGAGAtt	GUUCUUCAGUGACGCCCGAtt
SUMO1	CAAAGAAUCAUACUGUCAAtt	GAGAAUUGCUGAUAAUCAUtt	GACAGGGUGUUCCAAUGAAtt
SUMO3	AGGCAGGGCUUGUCAAUGAtt	GUGGUGCAGUUCAAGAUCAtt	CCAUCGACGUGUUCCAGCAtt
TERT	GCACGGCUUUUGUUCAGAUtt	GGCCGAUUGUGAACAUGGAtt	CGGAGACCACGUUUCAAAAtt
THAP1	CGACAAGGACAAGCCCGUUtt	AGGACAAGCCCGUUUCUUUtt	AUAUGUACAUAUUCUCAUAtt
TDG	GACGUAUUCUAGUACAGAAtt	CCGUUUUAAUGGUGUUUCAtt	UCAUUACCCUGGACCUGGAtt
KAT5	GGAGAAAGAAUCAACGGAAtt	GCAAGCUGCUGAUCGAGUUtt	GGACGGAAGCGAAAAUCGAtt
TOLLIP	GCUGUUGAUUUAAGGCACAtt	GACUCUUUCUAUCUCGAGAtt	GCACUGUGCAUGAUUCCGAtt
TOPBP1	GGAUAUAUCUUUGCGGUUUtt	GCAGAACUGUUGCGGAUUAtt	GCUCUGUAAUAGUCGACUAtt
TOP3A	CGGCUUGCCUAGUUCUCUAtt	CAGGUUAAAGUUAAAGUUUtt	GUGUACAGGUUAAAGUUAAtt
TOPORS	GUACAACUCUGACAAGGGAtt	GGAAAGUAGCAGACCUAGAtt	GAAAGAUCUUUGCGGAAAAtt
NR2C1	CCAUUGAAGUAUCACGAGAtt	GGUUUUUGAUCUUUGCGUAtt	GCGAUUCACAUGUAGCUUUtt
TRADD	CUGUUUGAGUUGCAUCCUAtt	GCGCAUACCUGUUUGUGGAtt	GAUGCGCUGCGAAAUCUGAtt
TERF1	ACCUAGUGGUAAUGAUGUUtt	GCAGAAUACCUGUUUCAAAtt	CAAAAGGACAAGAACAAUAtt
TERF2	GGUCGAAUCCAGUAGAAAAtt	GUACAACCAAUAUAACAAAtt	GAACAAGCGCAUGACAAUAtt
TRIM16	GCAUGGUGAAUUACUGUGAtt	GCAUCAGGUGAACAUCAAAtt	CUGCCUUGAUGACACCAGAtt
TDP2	GAUUAGAUCUAAACAAUCUtt	GAAUAACUGCUGCUUGUAAtt	GAAGAGAUCAAGUAAUUAUtt
UBA2	CGAAUGCAUCAGAUCAACAtt	CCCGAAAGCUAAUAUCGUUtt	GGAAACCUCCAGUUCCGUUtt
WRNIP1	CCAAGGCUGUCAUUUUAUAtt	GAGAUUCAUCGGUUCAAUAtt	GAAACAUAGCAUAAGGUUUtt
DAPK3	AGUUUGCGAUCGUGCGGAAtt	GAGGAGUACUUCAGCAACAtt	CCAACAUCUCAGCCGUGAAtt
ZMYM2	CAACAUUGCUUACUGCGUUtt	GUCUCACGAAUGUAGGAAAtt	GUGUGGAAGUAAUCGAAAAtt
ZNF451	CCGUCUAGAUGAACAACUAtt	GGAAAACUGUGGUUUCGCUtt	GCAUGCUCAUGGGUUACAAtt
ZNF506	GACCUUUGGUCAGAGCAGAtt	CCCGUACUACAUAUAAGAAtt	GUCUCUAAACCAAACCUGAtt
HIPK2	GAGAAUCACUCCAAUCGAAtt	CGGACUCACCAUAUCCUUUtt	CCAUGACCUUUAACAACCAtt
HIRA	GCUCCGAUCCUUCCAUGUAtt	CGAAGGGUUUGAAUACCGAtt	CGUCCAAGAUCGAACCCAUtt
CBX1	ACAGCACAUGAGACAGAUAtt	GCGCAAAGCUGAUUCUGAUtt	AGGAAUAUGUGGUGGAAAAtt
CBX3	GGAGAAUUGAUGUUUCUCAtt	GACGUGUAGUGAAUGGGAAtt	AGUACUAGAUCGACGUGUAtt
HSF2	CUCUGUAGAUAAACCCAUAtt	CUUGUGAGUUUAAAACGUAtt	GGAUUAUCUUGACAGUAUUtt
IKBKE	CGACACCAGGAGUACCUCUtt	CAGUGCUGUUUGGACAAGAtt	AGCUGGAUAAGGUGAAUUUtt
ISG20	AGCUGGUGGUGGGUCAUGAtt	AGAGAUCACCGAUUACAGAtt	CGAUGGAGCUCUAUCAAAUtt
JRK	ACACGGCAUUAAAAAGCUAtt	GCCGUUCUCUUGCUGGACAtt	GCAGAGUGCUUCCCAGUGAtt
NR5A2	GUGUUGGAAUGAAGCUAGAtt	CGACCACAUUUACCGACAAtt	GCACGGACUUACACCUAUUtt
MDM2	AGUCUGUUGGUGCACAAAAtt	AGACACUUAUACUAUGAAAtt	CUAUGAAAGAGGUUCUUUUtt
MORC3	GGAACAAAUUCUUAUGCGAtt	GAAUUACCUUUGGAUUCAAtt	CGCAAUCGUUUUUACCAAAtt
MRE11A	GAUAGACAUUAGUCCGGUUtt	CCCGAAAUGUCACUACUAAtt	CGACUGCGAGUGGACUAUAtt
MX1	CCAUCGGAAUCUUGACGAAtt	GAAGAAACUUGUGAACGAAtt	CAGAGGAGCUACAAAAGUAtt
MYB	GAAUUGCUCCUAAUGUCAAtt	GGAAAAGCGAAUAAAGGAAtt	CCUCUCAUCUAGUAGAAGAtt
N4BP1	CUGGUACAGUGUAUCCAGAtt	GGAAUACAACAUCCUCGUAtt	GGAGUCACAUUCAACAAUUtt
NCOA2	GAAUGGAUAUGAUUAAGCAtt	GAGCCUUAGGAAUACCCGAtt	CAGUCAAAUCUAUCGUUUUtt
CALCOCO2	CAUUGACCUAAACAACAAAtt	GGAGGAGCUAGAAACCCUAtt	CCUUCAUGUGGGUUACUUUtt
NFATC1	GGUCAUUUUCGUGGAGAAAtt	AGAUGGAAGCGAAAACUGAtt	GCCGGAAUCCUGAAACUCAtt
SLF1	GCAGGAAAGUGGAUACUAAtt	GCUGGAAAGGCAAAUGUUAtt	CCAGCCAUGUCGAGAUAUUtt
SLF2	GCUUUCAUGAAAGGUGUUAtt	GCAGAGAACGAGAACUAAAtt	GCCACCAUUCUACCAGGAAtt
RAD18	GGAAUCAUCUGCUGCAGUUtt	GCUCUCUGAUCGUGAUUUAtt	GCAUGGGACAGGAAGAUAAtt
RNF8	AGAAUGAGCUCCAAUGUAUtt	CAGAGAAGCUUACAGAUGUtt	UCAUUCAAGCCAAGAACAAtt
RNF168	GGACAUUGACAGUAGUGAUtt	CCUUGUGGCAGAACAGAAAtt	GCCAAAUUCUACUAGAGAUtt

Knockdown of SLF2 resulted in HBV-ΔX transcriptional activation in PHHs, excluding the possibility that SLF2 is a cell line-specific restriction factor for viral transcription (Fig. 2C and D). To establish whether SLF2 is involved in HBV transcriptional repression, we generated an SLF2 knockout single-cell clone based on the HepG2-NTCP cell line. Specifically, we introduced a 22-bp deletion in exon 3 of *SLF2*, resulting in early termination of protein translation (Fig. 2E). SLF2 knockout had no impact on cccDNA formation in the nucleus (Fig. 2F) but significantly increased the level of viral transcripts of HBV-ΔX compared with that in wild-type HepG2-NTCP cells (Fig. 2G). Moreover, the HBV transcription activated by SLF2 knockout was repressed upon ectopic expression of SLF2 in the cells (Fig. 2H). These results indicate that SLF2 is a host factor that represses HBV transcription.

SLF2 has been identified as a component of the DNA repair complex and is known to be responsible for the interaction of the SMC5/6 complex with DNA lesions (34). Knockdown of any subunit other than NSE2 of the SMC5/6 complex results in degradation of the whole complex (35). Our data showed that SLF2 knockout did not lead to any significant alteration in the protein levels of SMC5, SMC6, and NSE4 in the cells (Fig. 2I), ruling out the possibility that SLF2 knockout may affect the subunits’ stability of the SMC5/6 complex. Therefore, these results demonstrate that SLF2’s function in repressing HBV transcription is not dependent on regulating the stability of the SMC5/6 complex.

### SLF2 interacts with the SMC5/6 complex through residues 590 to 710, and its interaction with cccDNA requires the SMC5/6 complex.

**FIG 3:**
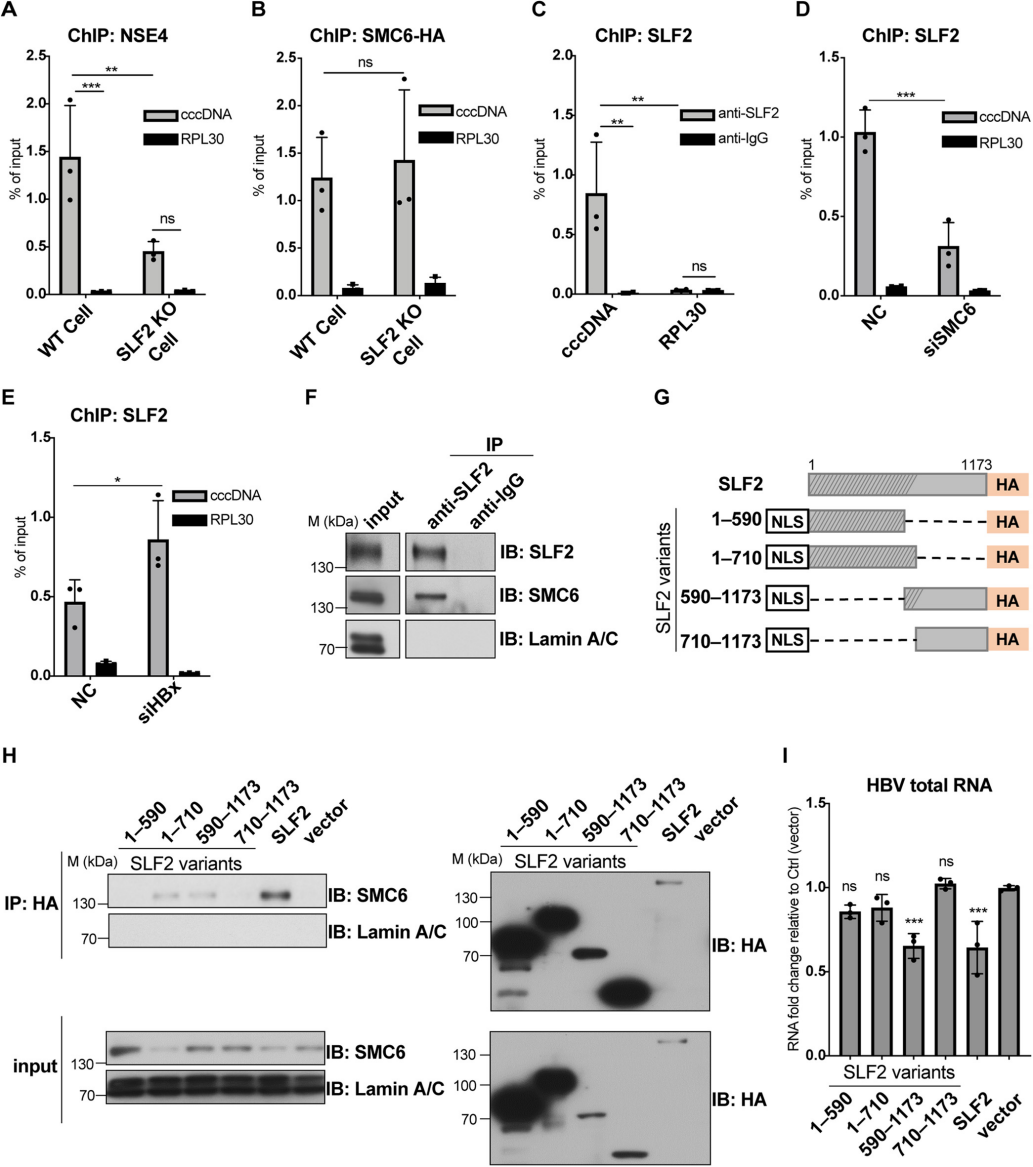
SLF2 interacts with SMC5/6 through residues 590 to 710, and its interaction with cccDNA requires SMC5/6. (A and B) Effect of SLF2 on the interaction between cccDNA and SMC5/6. WT or SLF2 KO HepG2-NTCP cells were inoculated with HBV-ΔX. Infected cells were fixed at 7 dpi and subjected to chromatin immunoprecipitation (ChIP). ChIP was performed using antibodies against NSE4 (A). RPL30 was taken as a negative control. Data from three independent experiments are presented as the percentage of input DNA. SLF2 KO or WT HepG2-NTCP cells were inoculated with HBV-ΔX and transduced with lentivirus-expressing SMC6-HA at 3 dpi. ChIP was performed using antibodies against HA at 8 dpi (B). (C) SLF2 interacts with cccDNA. HepG2-NTCP cells were inoculated with HBV-ΔX and treated in the same manner as in panel A. ChIP was performed using antibodies against SLF2 or IgG. (D and E) The SMC5/6 complex is required for the interaction of SLF2 with cccDNA. HepG2-NTCP cells were inoculated with HBV-ΔX and transfected with siRNA at 3 dpi. Cells were harvested at 8 dpi. ChIP was performed using antibodies against SLF2 (D). HepG2-NTCP cells were transfected with siRNA. After 48 h, cells were inoculated with HBV-WT. Cells were harvested at 3 dpi. ChIP was performed using antibodies against SLF2 (E). (F) SLF2 interacts with the SMC5/6 complex. HepG2-NTCP cells were cultured in PMM for 48 h, and the nuclei were separated and lysed. Nucleus lysates were subjected to SLF2 immunoprecipitation (IP). The SMC6 and SLF2 were analyzed by Western blotting. Lamin A/C was taken as a nucleus input indicator. (G and H) SLF2 interacts with SMC5/6 through residues 590 to 710. Full-length SLF2 cDNA was cloned from human liver cDNA. Truncated SLF2 was cloned with nuclear localization signal (NLS) and HA-tag. The shaded gray area indicates the predicted intrinsically disordered region (G). SLF2 knockout HepG2-NTCP cells were transduced with lentivirus-expressing SLF2 or truncation variants. Nucleus lysates were subjected to anti-HA IP. The SMC6 and SLF2-HA were analyzed by Western blotting (H). (I) Truncation variant SLF2-(590–1173) repressed HBV transcription. SLF2 KO HepG2-NTCP cells were inoculated with HBV-ΔX and transduced with lentivirus-expressing SLF2 or truncation variants at 3 dpi. Data of relative levels of HBV total RNA from three independent experiments are presented. The *P* value was calculated using two-way ANOVA (A to E) and one-way ANOVA (I). Error bar shows the SD. ns, not significant; *, *P* < 0.05; **, *P* < 0.01; ***, *P* < 0.001.

A previous study reported that the SMC5/6 complex interacts with HBV cccDNA (16). As SLF2 is implicated in the recruitment of the SMC5/6 complex to DNA lesions (34), it could promote the interaction between the SMC5/6 complex and cccDNA. To confirm this, we performed chromatin immunoprecipitation (ChIP) on HepG2-NTCP cells infected with HBV-ΔX and found that NSE4, a component of the SMC5/6 complex, precipitated cccDNA, validating the previously reported interaction between the SMC5/6 complex and cccDNA (Fig. 3A). Knockout of SLF2 considerably reduced the cccDNA levels precipitated by NSE4 (Fig. 3A), implying that SLF2 may be necessary for the interaction between the SMC5/6 complex and cccDNA. Intriguingly, when anti-SMC6-HA antibody was used in the ChIP assay, SLF2 knockout showed no effect on the SMC6-HA enrichment on cccDNA (Fig. 3B), consistent with a previous report that SLF2 is not required for the interaction of SMC5/6 with episomal DNA (36). SMC6 was shown to be presented exclusively in the SMC5/6 complex (35); hence, the observed discrepancy may be due to the loss of NSE4 binding in the absence of SLF2. Nonetheless, we found that the SLF2 antibody could precipitate HBV-ΔX cccDNA (Fig. 3C), and the precipitation efficiency significantly decreased when SMC6 was knocked down by siRNA (Fig. 3D). To determine whether SLF2 could interact with HBV-WT cccDNA when its transcription is silenced, we knocked down HBx to prevent the degradation of SMC5/6 in HBV-WT-infected cells. HBx knockdown effectively restored cccDNA precipitation by the anti-SLF2 antibody (Fig. 3E). Overall, these findings suggest that the SMC5/6 complex interacts with SLF2 and is necessary for interaction between SLF2 and cccDNA.

SLF2 is known to interact with the SMC5/6 complex (34, 37). To examine the interaction between SLF2 and the SMC5/6 complex in HepG2-NTCP cells, we precipitated SMC6 in the nucleus using an SLF2 antibody. SMC6 was pulled down by SLF2 in HepG2-NTCP cells, confirming an interaction between SLF2 and the SMC5/6 complex (Fig. 3F). The SLF2 protein comprises an intrinsically disordered N terminus (region 1 to 710) and a well-folded C terminus (region 710 to 1173) (37). To determine which region of SLF2 interacts with the SMC5/6 complex, we constructed four SLF2 truncation variants (Fig. 3G). SLF2-(590–1173) was aligned as the homologous region of the distant yeast ortholog (Nse6) (37), which was previously shown to recruit Smc5/6 to genomic DNA (38). SLF2-(1–590) was designed as the complementary variant of SLF2-(590–1173). We selected SLF2-(1–710) and SLF2-(710–1173) variants according to the predicted intrinsically disordered region (IDR) and well-folded region, respectively, as IDR has the potential for liquid-liquid phase separation (37, 39). After introducing these four truncation variants and full-length SLF2 into HepG2-NTCP cells, we found that SLF2-(1–710) and SLF2-(590–1173) variants could pull down SMC6, although not as efficiently as the full-length SLF2; no SMC6 pulldown was detected with the SLF2-(1–590) or SLF2-(710–1173) variant (Fig. 3H). These results collectively suggest that SLF2 may interact with the SMC5/6 complex through residues 590 to 710.

To evaluate the potential contribution of the SMC5/6-binding region (590 to 710) in viral repression, we examined the HBV repression function of SLF2 variants. We ectopically expressed SLF2 and its variants in SLF2 knockout HepG2-NTCP cells and found that, except for full-length SLF2, only the SLF2-(590–1173) variant could repress HBV transcription (Fig. 3I). These findings indicate that SLF2 represses cccDNA transcription through its interaction with the SMC5/6 complex. Interestingly, although SLF2-(1–710) does interact with the SMC5/6 complex, it does not exhibit repression activity (Fig. 3I), possibly due to the loss of the cccDNA interacting region.

### SLF2 recruits the SMC5/6 complex to PML bodies.

**FIG 4:**
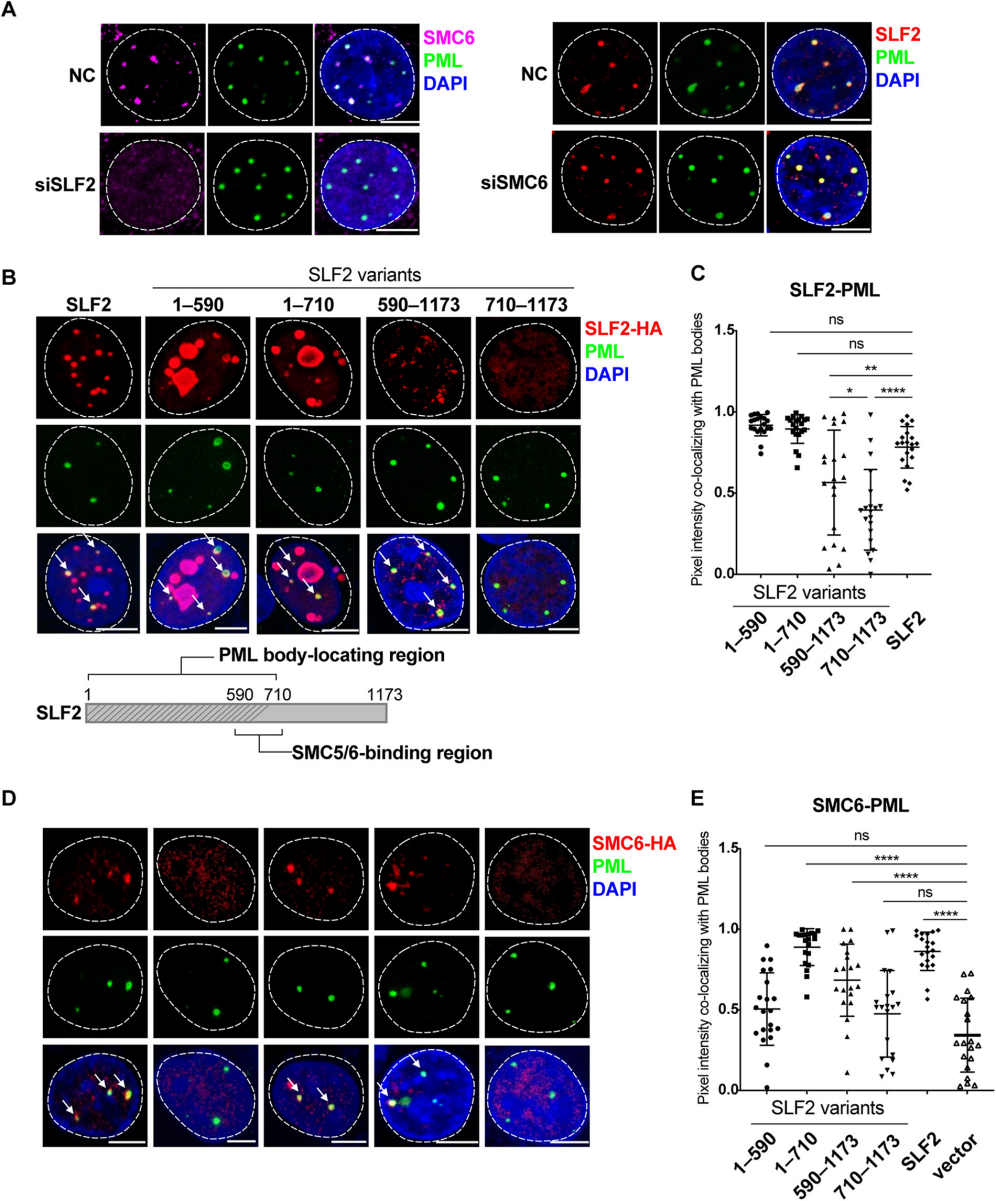
The intrinsically disordered region of SLF2 recruits SMC5/6 to PML bodies. (A) SLF2 directs SMC5/6 to PML bodies. PHHs were transfected with siRNA at 6 h after seeding and fixed after 72 h. Cells were stained for PML (green), SLF2 (red), or SMC6 (purple). (B and C) Colocalization of SLF2 with PML bodies is dependent on the intrinsically disordered region. SLF2 KO HepG2-NTCP cells were transduced with lentivirus-expressing SLF2 or SLF2 truncation variants and cultured in PMM for 48 h. Cells were fixed and stained for PML (green) and HA (red). The white arrows indicate the SLF2-HA foci colocalizing with PML bodies (B, upper panel). Schematic of the PML body-locating region of SLF2 (B, lower panel). The degree of colocalization was assessed by pixel intensity of SLF2-HA to PML staining signal with Fiji software. A total of 20 cells were analyzed in each group (C). (D and E) Recruitment of SMC5/6 to PML bodies is dependent on the disordered region of SLF2. SLF2 KO HepG2-NTCP cells were transduced with lentivirus-expressing SLF2 or SLF2 truncation variants and SMC6-HA and cultured in PMM for 48 h. Cells were fixed and stained for PML (green) and HA (red) (D). The white arrows indicate the SMC6-HA foci colocalizing with PML bodies. The degree of colocalization was assessed by pixel intensity of SMC6-HA to PML staining signal with Fiji software. A total of 20 cells were analyzed in each group (E). The *P* value was calculated using one-way ANOVA. Error bar shows the SD. The scale bar represents 5 μm. ns, not significant; *, *P* < 0.05; **, *P* < 0.01; ****, *P* < 0.0001.

The interaction of SLF2 with the SMC5/6 complex suggests that SLF2 may also colocalize with PML bodies. Immunofluorescence staining revealed that SLF2 was localized as nuclear foci and colocalized with SMC6 at PML bodies in PHHs (Fig. 4A). Knockdown of SMC6 did not affect SLF2 distribution, whereas knockdown of SLF2 disrupted the colocalization of SMC6 with PML bodies and dispersed it throughout the nucleus (Fig. 4A). Note that depletion of SLF2 did not reduce the protein level of SMC6 (Fig. 2I). These findings support that SLF2 can be plausibly conceptualized as an “anchor” that recruits the SMC5/6 complex to the PML bodies.

To identify which region of SLF2 is responsible for its colocalization with PML bodies, we expressed the wild-type or four SLF2 variants and examined their fluorescence signals’ colocalization with PML bodies. Full-length SLF2 formed speckle-like structures and colocalized with PML bodies (Fig. 4B and C). The SLF2-(710–1173) variant (lacking the intrinsically disordered region) did not form distinct foci in the nucleus (Fig. 4B), indicating that the IDR is essential for SLF2 concentration. Intriguingly, concentration capacity of the SLF2-(590–1173) variant containing part of the IDR (residues 590 to 710) was compromised, but this variant still colocalized with PML bodies (Fig. 4B and C). These results suggest that residues 590 to 710 of SLF2 promote its colocalization with PML bodies, and a longer IDR in the N terminus can enhance its ability to colocalize with PML bodies.

We next examined which region of SLF2 is necessary for recruiting SMC5/6 complex to PML bodies. Reexpression of full-length SLF2 restored SMC6 recruitment to PML bodies in SLF2 knockout HepG2-NTCP cells (Fig. 4D and E). However, neither the SLF2-(1–590) variant expressing the partial IDR nor the SLF2-(710–1173) variant expressing the well-folded C terminus restored the recruitment of SMC6 to PML bodies. Only the SLF2-(590–1173) or SLF2-(1–710) variant, which contains the SMC5/6-binding region, could recruit SMC6 to PML bodies (Fig. 4D and E). The variant [SLF2-(590–1173)] that recruited SMC6 to PML bodies also repressed HBV transcription (Fig. 3I), indicating that the colocalization of SLF2 and the SMC5/6 complex with PML bodies is necessary for HBV repression.

**FIG 5:**
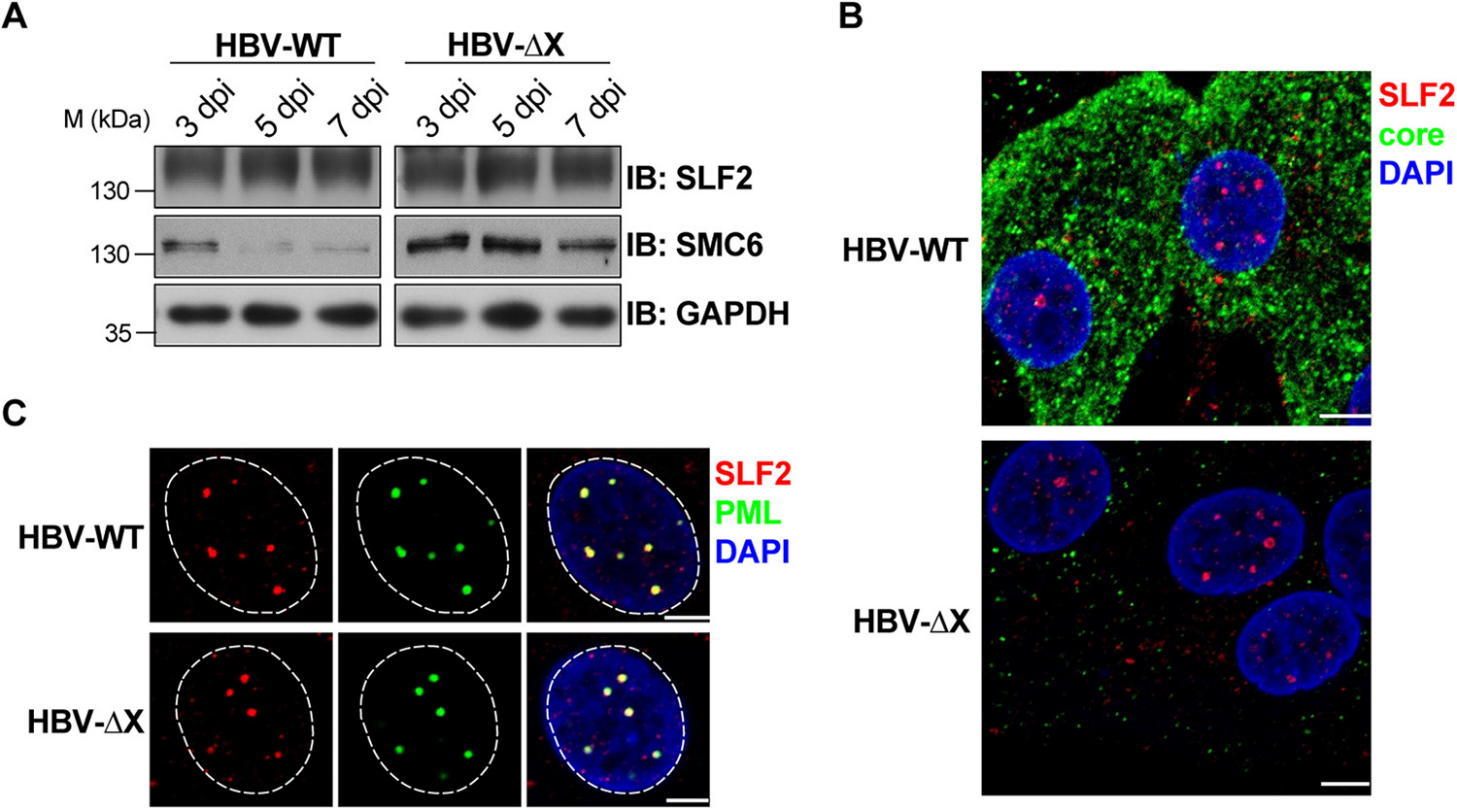
HBV infection has no effect on SLF2 stability and distribution. (A) No SLF2 degradation was induced by HBx. HepG2-NTCP cells were inoculated with HBV-WT or HBV-ΔX. Cells were harvested at the indicated times. Protein levels of SLF2 and SMC6 were examined by Western blotting. (B and C) PHHs were inoculated with HBV-WT or HBV-ΔX and fixed at 11 dpi. Cells were stained for core (green) and SLF2 (red) (B). Cells were stained for PML (green) and SLF2 (red) (C). The scale bar represents 5 μm.

Previous studies have reported that HBx leads to degradation of the SMC5/6 complex in HBV-infected cells (16). However, we found that the SLF2 protein level remained unchanged in both HBV-WT- and HBV-ΔX-infected cells (Fig. 5A). Furthermore, SLF2 distribution as nuclear foci and colocalization with PML bodies were unaffected by HBV infection (Fig. 5B and C), suggesting that HBV infection does not alter SLF2 stability and distribution.

### The colocalization of cccDNA with PML bodies is independent of the rcDNA repair process.

**FIG 6:**
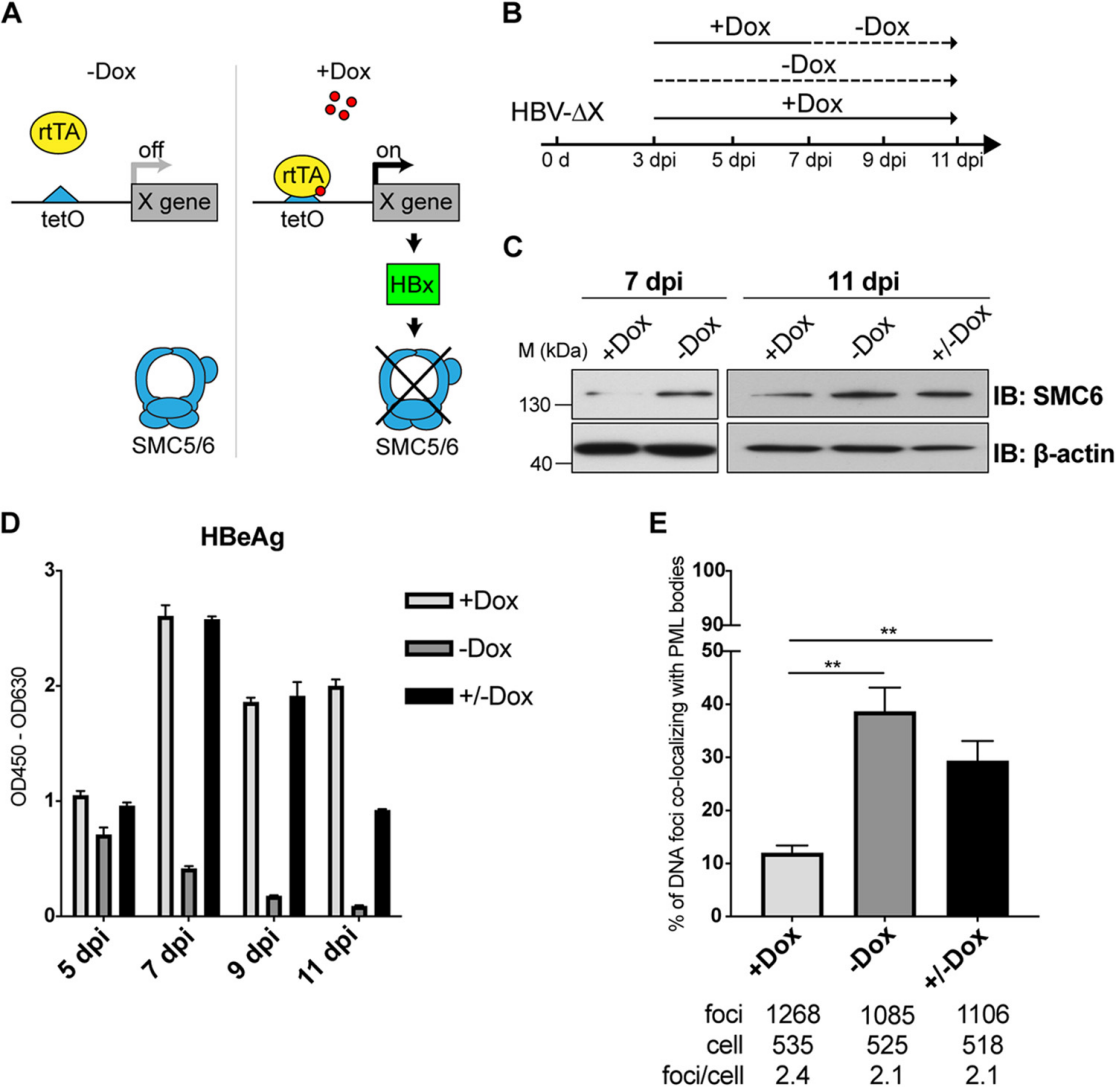
The colocalization of cccDNA with PML bodies is independent of the rcDNA repair process. (A) Schematic of the HBx Dox-inducible system. The HBV X gene was integrated into HepG2-NTCP cells (Tet-On control system). Cells expressed HBx, and the SMC5/6 complex was degraded with Dox treatment. (B to E) cccDNA positioned away from PML bodies with Dox treatment and colocalized with PML bodies without Dox. Cells were inoculated with HBV-ΔX and cultured with (i) medium with Dox from 3 dpi to 11 dpi, (ii) medium with Dox from 3 dpi to 7 dpi and Dox withdrawn from 7 dpi to 11 dpi, and (iii) Dox-free medium (B). The SMC6 protein (C) and HBeAg (D) were analyzed. Indicated samples were fixed at 11 dpi. HBV DNA was costained with PML, and the colocalization ratio of HBV DNA with PML was analyzed with the same method as in Fig. 1E (E). The *P* value was calculated using Fisher’s exact test. Error bar shows the SD (D) and SEM (E). **, *P* < 0.01.

HBV cccDNA is repaired from the rcDNA with an incomplete plus-strand, and host cellular DNA repair factors are involved in this process (5, 40). To investigate whether the repairing of rcDNA to cccDNA is linked to the colocalization of cccDNA with PML bodies, we used a Dox-inducible cell line to degrade the SMC5/6 complex. In brief, we induced HBx expression by administering Dox to HBV-ΔX-infected HepG2-NTCP cells harboring the HBx gene. These cells were infected with HBV-ΔX first, and the cells were then treated with Dox at 3 days postinfection when the cccDNA was formed in the nucleus. As anticipated, the protein level of SMC6 decreased and the HBeAg level increased upon Dox-induced HBx expression (Fig. 6A to D).

To evaluate the colocalization of cccDNA with PML bodies, we used DNA FISH and PML immunostaining. Our results demonstrated that HBx expression led to a decrease in the proportion of HBV DNA colocalizing with PML bodies. Moreover, withdrawing Dox resulted in the recovery of the proportion of HBV DNA colocalizing with PML bodies compared to the constant Dox-exposure group (Fig. 6E). These results indicate that the colocalization of cccDNA with PML bodies is predominantly independent of the rcDNA repair process.

### SLF2 directs cccDNA to PML bodies for HBV repression.

**FIG 7:**
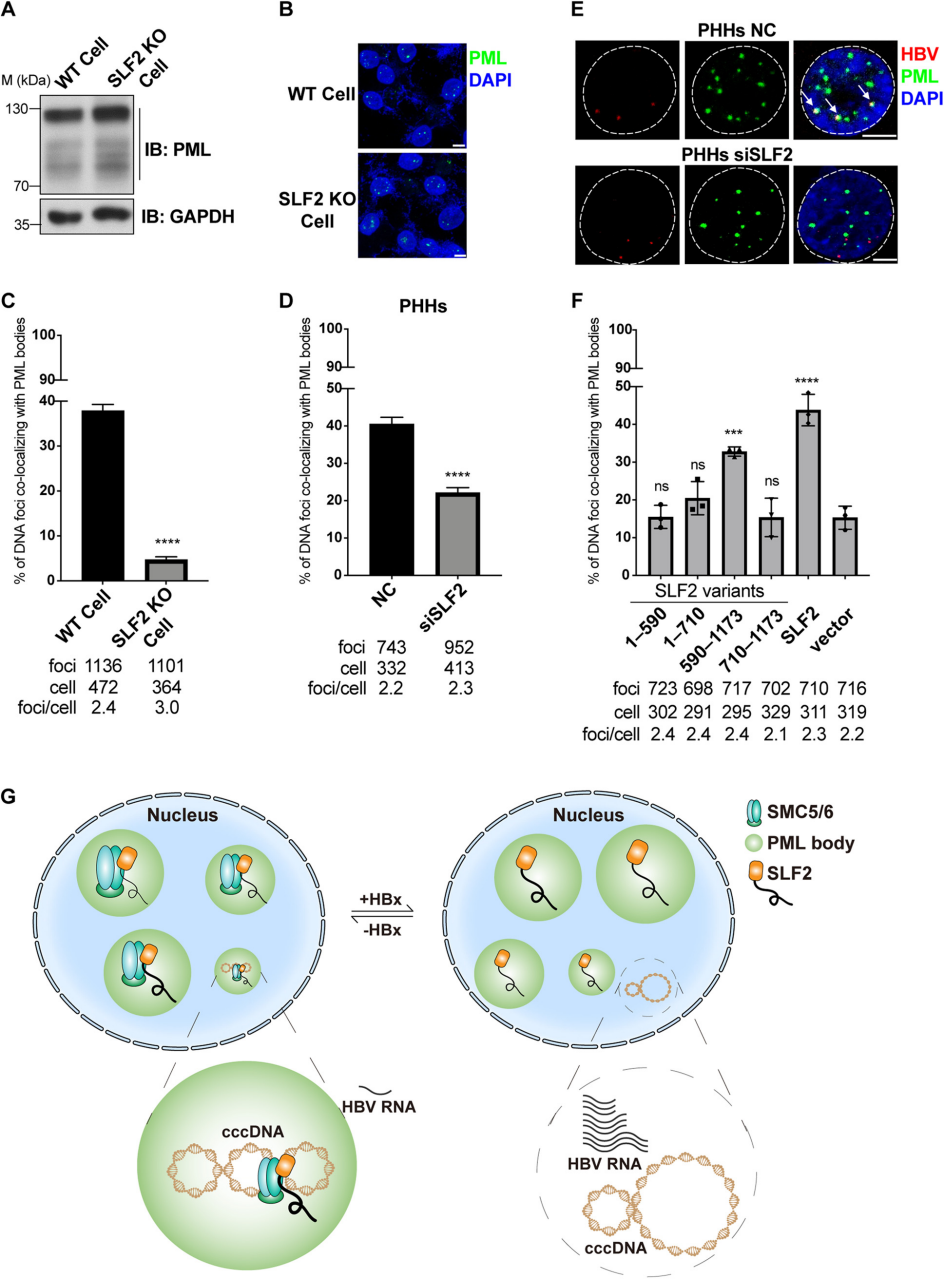
SLF2 directs cccDNA to PML bodies for HBV repression. (A and B) Knockout of SLF2 does not affect protein levels or the nuclear distribution of PML. WT or SLF2 KO HepG2-NTCP cells were treated with PMM for 48 h. PML proteins were analyzed by Western blotting (A). PML proteins were stained (B). (C) Knockout of SLF2 decreased the proportion of HBV-ΔX cccDNA colocalizing with PML bodies. WT or SLF2 KO HepG2-NTCP cells were inoculated with HBV-ΔX. Cells were treated with 5 μM HAP12 from 3 dpi to 7 dpi and fixed for DNA FISH at 7 dpi. The results are the summaries of three independent experiments. (D and E) Knockdown of SLF2 decreased the proportion of HBV-ΔX cccDNA colocalizing with PML bodies in PHHs. PHHs were transfected with siRNA at 6 h after seeding and then inoculated with HBV-ΔX at 16 h after siRNA transfection. Cells were treated with 5 μM HAP12 from 3 dpi to 11 dpi and fixed for DNA FISH at 11 dpi. (F) Effect of SLF2 truncation variants on the colocalization of cccDNA with PML bodies. SLF2 knockout HepG2-NTCP cells were inoculated with HBV-ΔX and transduced with lentivirus expressing SLF2 or SLF2 truncation variants at 3 dpi. Cells were treated with 5 μM HAP12 from 7 dpi to 10 dpi and fixed for HBV DNA FISH and PML staining. The colocalization of DNA foci with PML bodies was analyzed. (G) Schematic model of HBV cccDNA silencing mediated by SLF2 and the SMC5/6 complex. In the absence of HBx, SLF2 and the SMC5/6 complex interact with cccDNA and direct cccDNA to PML bodies. In the presence of HBx, the SMC5/6 complex is degraded, cccDNA locates away from PML bodies and viral transcription is activated. Orange rectangles represent the well-folded C terminus of SLF2, and the black curves represent the IDR of SLF2. The *P* value was calculated using Fisher’s exact test (C and D) and one-way ANOVA (F). Error bar shows the SEM (C and D) and SD (F). The scale bar represents 5 μm. ns, not significant; ***, *P* < 0.001; ****, *P* < 0.0001.

To investigate the role of SLF2 in the colocalization of cccDNA with PML bodies, we examined the proportion of cccDNA colocalizing with PML bodies in WT and SLF2 knockout HepG2-NTCP cells using DNA FISH. We found that knockout of SLF2 did not affect PML protein levels or the distribution of PML as nuclear foci (Fig. 7A and B). However, analysis of single HBV DNA foci in the nucleus revealed that knockout of SLF2 led to a significant decrease in the proportion of HBV-ΔX viral DNA foci colocalizing with PML bodies in HepG2-NTCP cells (Fig. 7C). This suggests that SLF2 promotes the colocalization of cccDNA with PML bodies. We confirmed this effect of SLF2 on PHHs by knockdown of SLF2, which also led to a decreased proportion of DNA foci colocalizing with PML bodies (Fig. 7D and E).

To determine whether the SLF2-mediated direction of cccDNA to PML bodies is essential for repression of viral transcription, we expressed the four SLF2 variants in SLF2 knockout HepG2-NTCP cells. We found that only expression of the SLF2-(590–1173) variant resulted in cessation of HBV-ΔX transcription (Fig. 3I). Moreover, only expression of the SLF2-(590–1173) variant led to the colocalization of cccDNA with PML bodies (Fig. 7F). These results suggest that the SLF2-mediated colocalization of cccDNA with PML bodies is crucial for repression of cccDNA transcription.

In summary, our findings suggest that SLF2 interacts with cccDNA in coordination with the SMC5/6 complex, directing cccDNA to PML bodies to repress viral transcription, and this host suppression is antagonized by HBx, which has no direct effect on SLF2 but leads to disruption of the SMC5/6 complex (Fig. 7G).

## DISCUSSION

In this study, we have presented evidence demonstrating the colocalization of transcriptionally silenced HBV cccDNA with PML bodies, indicating the involvement of PML bodies in the host restriction of HBV transcription. Furthermore, we have identified SLF2 as a host restriction factor for HBV transcription, which interacts with HBV cccDNA in the presence of the SMC5/6 complex and directs HBV cccDNA to PML bodies for transcriptional silencing. Our findings are consistent with previous studies suggesting that the SMC5/6 complex likely functions through PML bodies as a host restriction factor for HBV transcription.

Previous research has shown that SLF2 is a component of the DNA repair complex and interacts with SLF1 and RAD18. The RAD18-SLF1-SLF2 complex recruits the SMC5/6 complex to DNA lesions (34). However, our study found that knockdown of SLF2-related DNA repair factors, including RNF8, RNF168, RAD18, and SLF1, had no effect on HBV-ΔX transcription activation (Fig. 2A). Furthermore, in our studies of HBV-ΔX infection of HepG2-NTCP cells with inducible HBx expression, preformed cccDNA can be recognized and directed to PML bodies in the presence of SMC5/6 and SLF2. Thus, the suppression of cccDNA transcription is unlikely to be associated with SLF2-related DNA repair mechanisms. Instead, our results suggest that localization of cccDNA to PML bodies after *de novo* infection correlates with cccDNA transcription suppression.

PML bodies have been reported as mediators of intrinsic immunity, and they function in the regulation of innate immune signaling in host antiviral defense (26, 41). Viruses can alleviate this restriction by degrading proteins related to PML bodies or disrupting the whole structure of PML bodies (41). For example, the Vpr of HIV-1 was reported to target and degrade SLF2 to antagonize host restriction (37). In contrast, HBV infection does not affect the level or distribution of SLF2. Rather, HBx induces the degradation of SMC5/6. The interaction between the SMC5/6 complex and cccDNA indicates a direct function of the SMC5/6 complex in viral repression, as the SMC5/6 complex may repress viral transcription by compacting viral episomal DNA into a tightly folded minichromosome (37, 42). The repression mechanism of PML bodies for HBV silencing may also involve regulating epigenetic modifications of cccDNA (36, 43), as repressive epigenetic modifications are enriched on HBV-ΔX cccDNA compared to HBV-WT cccDNA (44, 45). However, it is currently unclear how these mechanisms, such as SMC5/6-mediated cccDNA minichromosome compaction, epigenetic modifications of cccDNA, and potentially other yet-to-be-revealed mechanisms jointly contribute to the repression of HBV transcription.

In a recent study, it was reported that the SMC5/6 complex can silence episomal transcription through a three-step function and that SLF2 is essential for the interaction of PML protein with a reporter episomal DNA (36). This finding supports our research, which shows that SLF2 is responsible for directing HBV cccDNA to PML bodies and that knockout of SLF2 significantly decreases the proportion of HBV-ΔX cccDNA colocalizing with PML bodies. In our study, we also discovered that the binding capacity of the SMC5/6 complex with cccDNA, examined using an NSE4 antibody, was considerably reduced in SLF2 knockout cells, suggesting that SLF2 is necessary for the interaction of the SMC5/6 complex with cccDNA. This result is consistent with a study conducted on HIV, which also found that SLF2 is required for the interaction of SMC5/6 with viral DNA, as measured by the HA-NSE2 subunit (37). However, the binding between HA-SMC6 and episomal DNA remained unchanged in SLF2 knockout cells (36). It is possible that the NSE2 and NSE4 subunits are inaccessible in the absence of SLF2 due to the conformational change of the complex. Future structural studies may elucidate the details of SMC5/6 complex binding with HBV cccDNA.

Through HBV cccDNA FISH and imaging analysis, we determined the proportion of cccDNA that colocalizes with PML bodies. We found that approximately 50% of HBV-ΔX cccDNA colocalizes with PML bodies in PHHs. Although a significant proportion of HBV-ΔX cccDNA is not in proximity to PML bodies, the transcription of these HBV-ΔX cccDNAs is minimal as assessed by enzyme-linked immunosorbent assay (ELISA) and Northern blot analysis. This indicates that HBV-ΔX cccDNA that does not colocalize with PML bodies is also transcriptionally repressed. The apparent discrepancy between the transcriptional repression status of HBV-ΔX and the colocalization rate of cccDNA with PML bodies may be due to the dynamic assembly and disassembly of PML bodies in the nucleus (46).

Previously, we reported that HBV-ΔX cccDNA tends to aggregate at transcriptional silencing compartments of chromosome 19, and depletion of SMC6 reduces this aggregation based on chromosome conformation capture-on-chip (4C) analysis and chromosome/HBV FISH studies (19). This suggests that, in addition to PML bodies, the interaction between the SMC5/6 complex and cccDNA results in differential subnuclear positioning. It is possible that further investigation of the positioning of cccDNA relative to the host genome and nuclear bodies will provide a more complete understanding of the antiviral mechanism. Nonetheless, the SLF2-directed localization of HBV cccDNA to PML bodies for transcriptional repression represents a critical host defense mechanism and provides further support for antiviral drug development targeting an HBx related pathway.

## MATERIALS AND METHODS

### Cells, antibodies, and reagents.

Human hepatoblastoma HepG2 cells and human embryonic kidney 293FT cells were obtained from American Type Culture Collection (ATCC). Human hepatocellular carcinoma cells (Huh-7) were obtained from the Cell Bank of Type Culture Collection, Chinese Academy of Sciences. HepG2 and Huh-7 cells were cultured in Dulbecco modified Eagle medium (DMEM) with 10% fetal bovine serum (FBS), 100 U/mL penicillin, 100 μg/mL streptomycin, and 1× GlutaMAX (Gibco) at 37°C in a 5% CO_2_ incubator. The 293FT cells were cultured in DMEM with 10% FBS, minimal essential medium (MEM) non-essential amino acids (NEAA), and 1 mM sodium pyruvate (Gibco). PHHs were purchased from Shanghai RILD, Inc. (catalog [cat.] no. 00995). Antibodies for human SLF2 (ab122480), PML (ab179466, ab96051), and lamin A/C (ab238303) were purchased from Abcam. Antibodies for human SMC6 (AT3956a) and NSE4 (AP9909A) were purchased from Abcepta. Antibodies for hemagglutinin (HA; no. 3724) were purchased from Cell Signaling Technology. Horseradish peroxidase (HRP)-conjugated anti-glyceraldehyde-3-phosphate dehydrogenase (GAPDH) antibody, anti-mouse IgG, and anti-rabbit IgG were purchased from EasyBio. Alexa Fluor 488-, 546-, and 647-conjugated anti-mouse/rabbit IgG were purchased from Thermo Fisher Scientific. A mouse antibody against the HBV core protein (1C10) was obtained as previously described (6). HAP12 was synthesized according to a previously outlined molecular structure (33).

### Plasmids, small interfering RNAs, and transfection.

The single guide RNA (sgRNA) expression plasmid was constructed by inserting sgRNA into the pLKO.1-U6-trRNA-puro plasmid. The SMC6-expressing plasmid was purchased from Addgene. The full-length SLF2 cDNA was cloned from human liver cDNA. To construct the truncated NLS-SLF2-HA expression plasmid, an N-terminal nuclear localization signal (NLS) and a C-terminal HA tag were fused to the truncated SLF2 variants. For siRNA-mediated gene knockdown, siRNAs were transfected with Lipofectamine RNAi-MAX (Thermo Fisher Scientific). For gene knockdown in HepG2-NTCP cells infected with HBV-ΔX, the siRNA was transfected at 3 days postinfection (dpi). For gene knockdown in PHHs, cells were transfected with siRNAs at 6 h after cell seeding. After 16 h of incubation, siRNAs were removed, and cells were inoculated with HBV. For HBx knockdown in HBV-WT-infected HepG2-NTCP cells, the siRNA was transfected at 48 h before infection. The sequence of siRNA targeting HBx transcripts is 5′-CUGUAGGCAUAAAUUGGUCtt. Other siRNAs used in this paper were designed using an online RNA interference (RNAi) designer (https://rnaidesigner.thermofisher.com/rnaiexpress/) and are listed in Table 1. The gene knockdown efficiency was measured by quantitative PCR (qPCR).

### Virus production and *in vitro* viral infection.

Wild-type HBV (HBV-WT) was produced by transient transfection of Huh-7 cells with a plasmid harboring 1.05 copies of HBV genome (genotype D) as previously described (6). For X-minus HBV (HBV-ΔX) production, a stop codon mutation was introduced to the 8th codon in the HBx coding sequence. A plasmid expressing the wild-type HBx was cotransfected with the HBV-ΔX genome at a 1/3 molecular ratio to produce the X-minus virus. To generate pseudotyped lentivirus-expressing SMC6-HA and truncated NLS-SLF2-HA, plasmids were cotransfected with pSPAX2 and pMD2G into 293FT cells at a ratio of 4:3:1 with Lipofectamine 3000 (Thermo Fisher Scientific). Supernatant was collected at 48 h after transfection, filtered with a 0.22-μm filter, and stored at −80°C.

### Southern blot and Northern blot analysis.

HBV cccDNA was extracted by a modified Hirt method as previously described (47, 48). Infected cells were lysed in Hirt lysis buffer (10 mM Tris-HCl, 10 mM EDTA, 0.6% SDS, pH 7.4) for 30 min at room temperature. The cell lysate was mixed with 5 M NaCl and incubated at 4°C overnight. After centrifugation at 12,000 rpm for 30 min at 4°C, the supernatant was extracted twice with DNA extraction solution (phenol:chloroform:isoamylol, 25:24:1). The extracted DNA was precipitated with equal volumes of isopropanol with centrifugation at 12,000 rpm for 60 min at 4°C and was dissolved with TE buffer. The extracted HBV cccDNA was separated by 1% agarose gel electrophoresis and transferred onto Amersham Hybond-N^+^ membrane (Cytiva). The Hybond-N^+^ membrane was cross-linked in a UV cross-linker chamber and hybridized with digoxigenin (DIG)-labeled full-length HBV genome probe. After washing, the membrane was detected using the DIG-High Prime DNA labeling and detection starter kit II (Roche).

To detect HBV transcripts by Northern blotting, cells were harvested using TRIzol reagent (Thermo Fisher Scientific). The cell lysate was extracted with chloroform, and the extracted RNA was precipitated by isopropanol. Then 7 μg RNA was separated by 1.2% agarose gel electrophoresis and transferred onto Hybond-N^+^ membrane, and the membrane was hybridized with digoxigenin (DIG)-labeled full-length HBV genome probe and GAPDH probe. The GAPDH probe was amplified with the following primers: GAPDH-forward (F): 5′-GAAGGTGAAGGTCGGAGTCA; GAPDH-reverse (R): 5′-TTCCACGATACCAAAGTTGTC. After washing, the membrane was detected with the same method as in Southern blotting.

### SLF2 knockout cell line construction.

The SLF2 knockout cell line was constructed based on a cell line stably expressing Cas9 (HepG2-NTCP-Cas9). sgRNA targeting SLF2 (5′-GAACTGGAATTTAGCTCCCAG) expressing lentivirus was transduced into HepG2-NTCP-Cas9 cells. Cells were then cultured in the medium supplemented with 5 μg/mL blasticidin and 2 μg/mL puromycin selecting for expression of Cas9 and sgRNA. The genomic sequence around the sgRNA targeting site of a single-cell clone was amplified (F: 5′-ACCACAGTGGCAGAAGCTGACATC; R: 5′- GCTTCTGAGAAAGAATTCTCGGAG) and ligated to the T-vector plasmid. Nucleotide editing was examined by Sanger sequencing. The protein expression level was examined by Western blotting.

### Coimmunoprecipitation.

To capture the interaction between SLF2 and the SMC5/6 complex, HepG2-NTCP cells (2 × 10^6^ cells) were cultured in PHH maintenance medium (PMM) for 48h, and cells were then digested by Accutase (Thermo Fisher Scientific) and resuspended with DMEM (with 10% FBS). Cells were washed with prelysis buffer (10 mM Tris-HCl, 10 mM NaCl, 3 mM MgCl_2_, pH 7.4). Then cells were lysed with cell lysis buffer (0.1% CA-630, 1× cocktail, 10 mM Tris-HCl, 10 mM NaCl, 3 mM MgCl_2_) on ice for 10 min and treated with Dounce homogenizer for 50 strokes to disrupt the cell membrane. The nuclei were collected by 1,400 × *g* centrifugation for 5 min. The nuclei were lysed with nuclei lysis buffer (1% CA630, 1× Tris-buffered saline (TBS), 1:500 benzonase, 1× cocktail) for 15 min on ice. The supernatant was collected for protein immunoprecipitation after 10,000 × *g* centrifugation. Dynabeads Protein A (Thermo Fisher Scientific) was incubated with SLF2 antibody or anti-rabbit IgG in phosphate-buffered saline (PBS; with 0.02% Tween 20) for 20 min at room temperature. The cell lysate was incubated with beads-Ab complex for 3 h at 4°C. The Dynabeads-Ab-Ag complex was washed 5 times with washing buffer (1× TBS, 1× cocktail, 250 mM NaCl). Protein was eluted using elution buffer (2% SDS, 50 mM Tris, pH 8) for 15 min at 70°C. Eluted samples were analyzed by Western blotting. To analyze the interaction region of SLF2 with the SMC5/6 complex, lentiviruses expressing truncated SLF2 variants were transduced into SLF2 knockout (KO) cells, and cells were supplemented with fresh PMM 24 h posttransduction. Cells were then digested and subjected to co-immunoprecipitation IP (co-IP) as previously described.

### ELISA for HBV antigen.

The HBeAg and HBsAg in the supernatant of HBV-infected cells were measured using ELISA kits (Wantai BioPharm, Inc. Beijing, China) according to the manufacturer’s instructions.

### Chromatin immunoprecipitation-qPCR (ChIP-qPCR).

The interaction between protein and DNA was measured with a SimpleChIP enzymatic chromatin IP kit (Cell Signaling Technology). In brief, the cells were fixed with 1% formaldehyde for 10 min, and the glycine was then added to terminate the cross-linking. The cells were scraped from the dish and collected using cold PBS (with proteinase inhibitor). After centrifugation, cells were resuspended with lysis buffer A and lysis buffer B for nucleus preparation. Lysate of 4 × 10^6^ cells was supplemented with micrococcal nuclease to digest DNA at 37°C for 20 min. Digestion was stopped by 50 mM EDTA, and nuclei lysates were collected by centrifugation. The pellet was resuspended with ChIP buffer and sonicated by 12 pulses (5s on, 5s off) at 20% amplitude using an ultrasonic processor. Next, 1/50 of lysates was removed as input sample. The remaining supernatant was incubated with antibodies against NSE4, SLF2, IgG, or histone 3 (positive control) at 4°C overnight. Samples were then incubated with ChIP-grade protein G magnetic beads for 2 h at 4°C. Protein-DNA on beads was eluted at 65°C. HBV cccDNA was quantified by the following primers: cccDNA-qF, 5′-GTGCACTTCGCTTCACCTCT; cccDNA-qR, 5′-AGCTTGGAGGCTTGAACAGT. The RPL30 exon3 qPCR primers were used as DNA enrichment control. The DNA enrichment was calculated as percent input = 2% × 2^(c[T] 2%Input Sample – C[T] IP Sample)^.

### DNA FISH and confocal microscopy.

HBV DNA FISH was performed as previously described (19). The FISH probes targeting the HBV negative strand were directly synthesized as oligonucleotides and mixed. The fluorescent secondary probe was modified at the 5′ and 3′ ends with an Alexa Fluor-647 dye. HepG2-NTCP cells were inoculated with HBV and reseeded to a collagen-coated coverslip at 3 dpi and treated with 5 μM HAP12 from 3 dpi to 7 dpi to inhibit rcDNA formation. After DNA FISH hybridization and washing, the sample was blocked with 3% BSA and subjected to antibodies against PML (1:500) for immunostaining. All images were captured using the Zeiss LSM800 confocal microscope with z-stack scanning (a total of 11 to 13 slices were captured with 0.5-μm step size).

### Colocalization analysis using Imaris software.

Confocal images of HBV DNA foci and PML bodies were analyzed with Imaris software (version 9.5.0, Oxford Instruments). Each HBV DNA foci was defined as a spot (diameter, 0.4 μm; quality, ≥20). The PML staining region was defined as an area (smooth, 0.2; absolute intensity, ≥20). The shortest distance (*d*) from each center of the DNA spot to the boundary of the PML body area was calculated by the software. The DNA colocalizing with PML was defined as *d* ≤ 0 μm. The colocalization ratio was calculated as the DNA number colocalizing with PML bodies/total DNA number. At least 200 cells from two or three independent experiments were analyzed.

### Colocalizing pixel intensity analysis.

The single channel of the HA or PML immune fluorescence image to be analyzed was transferred to a 16-bit type picture in Fiji software. Then the pixel intensity of HA colocalizing with PML was calculated using the “co-localization” function. The value of Manders’ M2, which is defined as the ratio of the “summed intensities of pixels from the PML image for which the intensity in the SLF2-HA channel is above zero” to the “total intensity in the PML channel” was analyzed (49).

### Immunofluorescence assay.

HepG2-NTCP cells were fixed with 3.7% paraformaldehyde for 10 min and washed with PBS 3 times. After permeabilization with 0.5% Triton X-100 for 10 min, cells were blocked with 3% BSA and incubated with antibodies against PML, SMC6, or SLF2 for 1 h at 37°C. After PBS washing, samples were incubated with fluorescent secondary antibodies for 1 h at 37°C. Images were captured using a Zeiss LSM800 confocal microscope.

### RNA-seq.

For the RNA sequencing (RNA-seq), 4 × 10^6^ WT or SLF2 knockout HepG2-NTCP cells were cultured in PMM for 48 h, and RNA was extracted using TRIzol reagent. Extracted RNA was dissolved in diethyl pyrocarbonate (DEPC)-treated water and subjected to RNA-seq. mRNA was enriched by poly(dT) probes and fragmented. Sequencing data were aligned to the GRCh38 reference genome using the Subread aligner. The bigWig files were visualized using the Gviz package in the R environment.
